# Recent advances in solid–liquid triboelectric nanogenerator technologies, affecting factors, and applications

**DOI:** 10.1038/s41598-024-60823-y

**Published:** 2024-05-07

**Authors:** Zhuochao Yuan, Lin Guo

**Affiliations:** Energy Research Institute, Qilu University of Technology, Jinan, 250014 China

**Keywords:** Energy harvesting, Triboelectric nanogenerators, Water–solid interface, Mechanical engineering, Electronic devices

## Abstract

Converting dispersed mechanical energy into electrical energy can effectively improve the global energy shortage problem. The dispersed mechanical energy generated by liquid flow has a good application prospect as one of the most widely used renewable energy sources. Solid–liquid triboelectric nanogenerator (S–L TENG) is an inspiring device that can convert dispersed mechanical energy of liquids into electrical energy. In order to promote the design and applications of S–L TENG, it is of vital importance to understand the underlying mechanisms of energy conversion and electrical energy output affecters. The current research mainly focuses on the selection of materials, structural characteristics, the liquid droplet type, and the working environment parameters, so as to obtain different power output and meet the power supply needs of diversified scenarios. There are also studies to construct a theoretical model of S–L TENG potential distribution mechanism through COMSOL software, as well as to obtain the adsorption status of different kinds of ions with functional groups on the surface of friction power generation layer through molecular dynamics simulation. In this review, we summarize the main factors affecting the power output from four perspectives: working environment, friction power generation layer, conductive part, and substrate shape. Also summarized are the latest applications of S–L TENG in energy capture, wearable devices, and medical applications. Ultimately, this review suggests the research directions that S–L TENG should focus on in the future to enhance electrical energy output, as well as to expand the diversity of application scenarios.

## Introduction

Since the field of triboelectric nanoelectricity generation was proposed in 2012, the concept of multiphase coupled triboelectric nanoelectricity generation has been proposed along with it. The electrical signals generated by friction nanogeneration are different from the traditional magneto-generated electricity, which has the characteristics of small current, low frequency and high voltage^1^. Currently, triboelectric nanogenerators is dominated by solid–solid triboelectric nanogeneration, which has been widely studied because it has the most obvious electrical signal characteristics compared with solid–liquid, liquid–liquid and solid–gas phase triboelectric nanogeneration. However, solid–solid triboelectric nanoelectricity generation has the problems of short generation duration and the need of external friction on dielectric materials. Solid–liquid triboelectric nanogeneration avoids these defects and it not only has the advantages of low friction between two phases, long power generation time, self-generation and self- cleaning, but also shows high research value due to the strength of the electrical signal is only second to the solid–solid friction nanogeneration^2^.

Numerous reviews summarized the progress in the field of triboelectric nanogenerator. Concerning the interfacial surface of TENG, Sun et al. reviewed the working structure of interface, which was classified into one-, two-, three-dimensional interface and noncontact interface, and summarized each interface in design principle, processing, performance and application^2^. Interfacial material of TENG was studied, such as superwetting surface was reviewed for its advantage, progress and application (i.e. self-powered sensors, self-powered anticorrosion and antibiofouling, wearable and implantable power generation, hybrid power generator, and ocean energy collector)^3^. Other interfacial materials were studied as well. For instance, low-dimension carbon material (e.g. graphene and carbon nanotube) were inexpensive and stable, its application could improve TENG is flexibility, transparency, and stretchability^4^. From the perspective of chemical functionalization, interfacial material was reviewed for the effects of solid surface charge density, wetting property and liquid property on solid–liquid electrification^5^. The above review also covered the impact of chemical functional groups on hydrophobicity and surface charge density of solid materials, as well as electrification efficiency of liquid materials^5^. The overall progress of TENG, including theory, materials, devices, systems, circuits, and applications, were summarized by Lin et al.^6^.

Concerning the energy source, liquid-based triboelectric nanogenerator (L-TENG) refers to a triboelectric nanogenerator with liquid involved in the power generation process. L-TENG included S–L TENG and water-TENG. Tang et al. introduced four working mechanisms of S–L TENG (contact-separation mode, sliding mode, single-electrode mode and freestanding mode), as well as the application of L-TENG were (self-powered liquid sensors, wearable power generation, hybrid energy harvester and blue energy harvester)^7^. Water-TENG refers to those TENG which uses ocean energy (such as waves) to generate electricity. Summaries regarding water-TENG included its working mechanism, parameter of electrical signal, and application as self-powered sensor or actuator^8^, as well as its structural design^9^.

**Figure 1:**
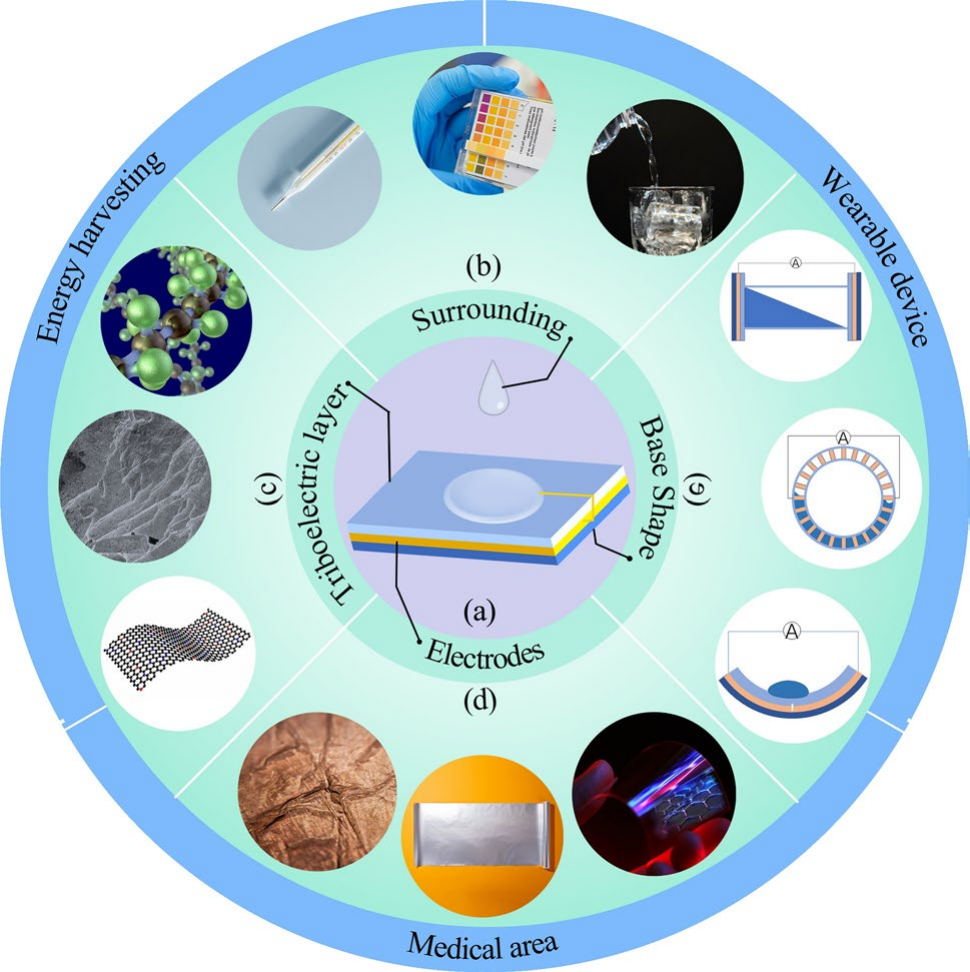
Overview of techniques, influencing factors, and applications of the solid–liquid triboelectric nanogenerator (S–L TENG). (a) Shows the basic structure of the S–L TENG. (b), (c), (d) and (e) shows the influencing factors. The rest of the outermost section summarizes the areas of application of S–L TENG.

Despite previous inclusive reviews of TENG from various perspectives, current publications lack a summary of the S–L TENG structure with a longitudinal categorization Considering the variety of S–L TENG, we categorized the relevant publications based on the structure of S–L TENG-triboelectric layer, conductive part and substrate in hope of facilitating the optimization of each module. In each module, we further focused on the means of enhancing the charge output. Specifically, this review introduced the working principle of S–L TENG, the influence of different friction droplets related parameters, temperature and different modules on energy output, and the S–L TENG use in a variety of areas is provided (Fig. 1). We hope to provide information on the overall field of S–L TENG and propose future research directions.

## Power generation efficiency influencing factors

Positive and negative charges in an insulator (dielectric material) repel each other under action of an external electric field and they are uniformly distributed on the upper and lower surfaces of the insulator. In this way, the insulator cannot transfer charges and therefore becomes non-conductive, which is the well-known polarization phenomenon.

The entire triboelectric nanogeneration process can be regarded as a charging stage and a steady power generation phase. In the charging stage, polarization occurs in insulators under continuous action of droplets because of the presence of positive and negative charges in the droplets. In the process, electrical signals appear out of nothing and become increasingly intense, finally being maintained at a peak, which is a slow process. Taking the droplet-based electricity generator (DEG) introduced by Xu as an example, the process from generation of electrical signals from nothing to reaching the peak calls for 1.6 × 10^4^ water droplets to continuously drop onto the triboelectric layer. Some studies suggest the use of pre-charge through electrowetting-assisted charge injection in droplets dropped^10^; injecting ions using a commercial antistatic gun (Zerostat3 and Milty) from the distance of 50 mm^11^; heating the entire system to reduce the charging time while applying high-voltage current between the needle and aluminum electrodes for coronal charging^12–15^. These methods can shorten the charging time. After the electret on the triboelectric layer is completely polarized, the charging process ends, when the electrical signals output has peaked.

In the process, the type and dropping angle of liquids needed for triboelectrification, materials of the triboelectric layer, surface structure, selection of electrodes and conducting parts, and even the overall shape of S–L TENGs affects the final characterization of electrical signals. These are analyzed in detail below.

### Influences of surrounding conditions on energy harvesting

#### Droplet effects on energy harvesting

**Figure 2:**
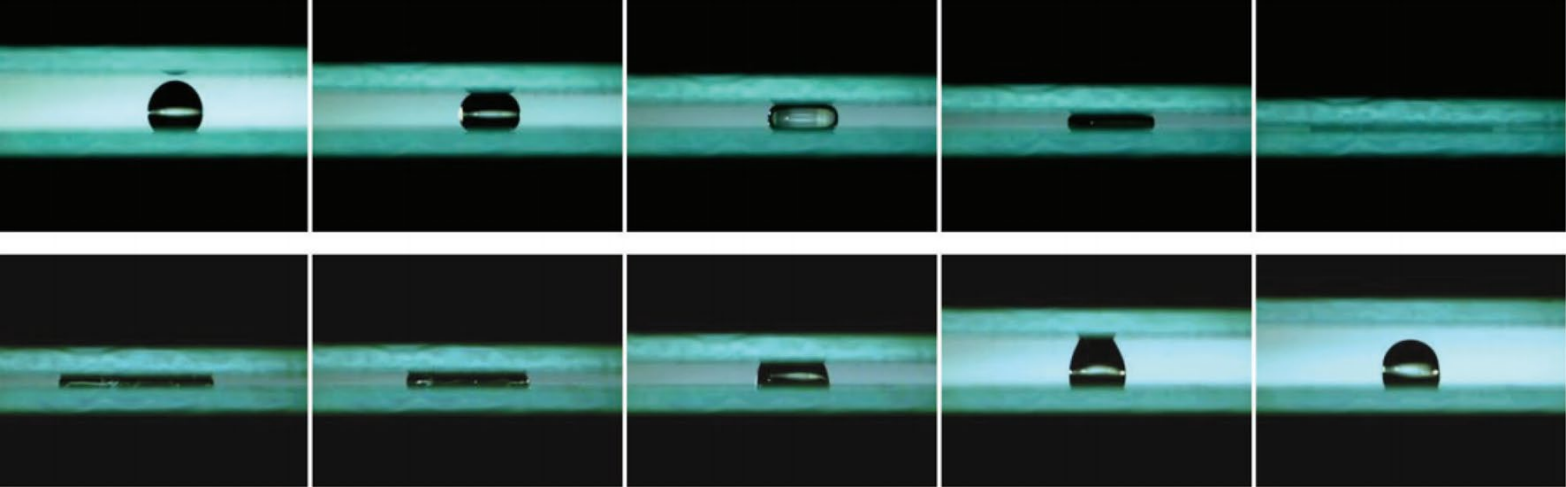
Extrusion of droplets by two substrates^17^.

Droplets, as the triboelectrification source of TENGs, are also considered when exploring the energy-harvesting principle of S–L TENGs. The solution concentration, pH value, dropping angle, number of charges, and number of ions in droplets all affect the experimental results. In the initial research, the contact area between droplets and triboelectric layers was controlled using a vertical contact-separation (CS) mode^16^. The process was generally executed manually, which imperceptibly induced some uncertainties to the experimental results. The distance between two substrates becomes more controllable with the introduction of the vertical motor, and the droplets are extruded more approximately as a thin film^17^ (Fig. 2).

When applying different concentrations of CuSO_4_ and NaCl solutions as triboelectric droplets, the number of transferred charges tends to increase, then decrease with increasing solute concentration of the solutions. Thereafter, the relationships of OH^−^ and H^+^ concentrations in droplets with charge transfer were studied using two materials, HCl and NaOH, which yields similar results as previous research. That is, the number of transferred charges always increases, then decreases with increasing ion concentrations in the solutions. Such a phenomenon is only observed in acid, alkali, and salt solutions, while not in deionized water. The review therefore concludes that if there are a small number of free ions transferred in solutions, it promotes the S–L triboelectrification; if the ion concentration is too large, ions are attached onto the triboelectric layer, inducing the shielding effect that hinders charge transfer^17–19^. In addition, electrical signals in pure water are better than those in tap water and NaCl solution.

**Figure 3:**
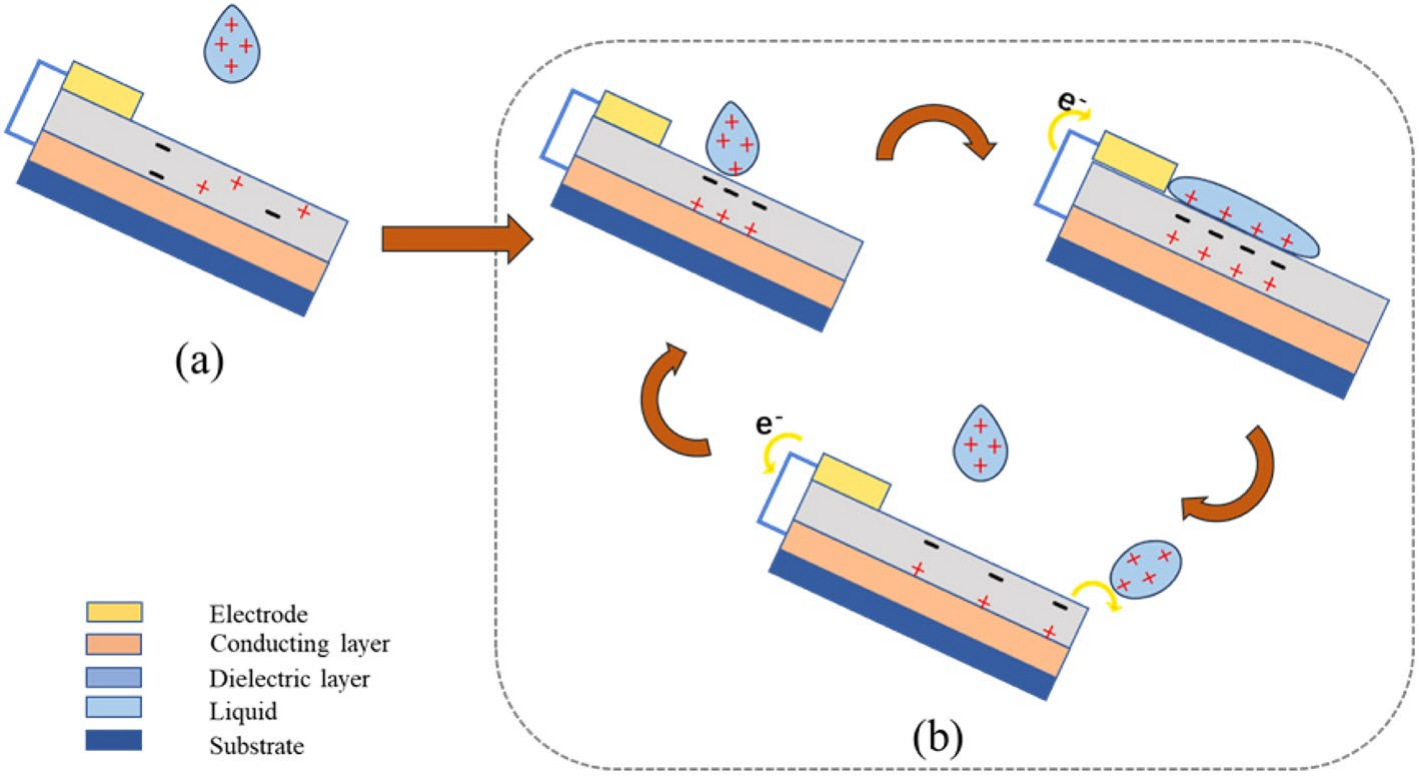
Theoretical modeling of the charge distribution mechanism in the process of droplet triboelectricity nanogeneration. The process (**a**) to (**b**) is the polarization of the electrons, the process will have a certain increase in potential eventually maintaining the peak output.

The droplet and charge-transfer process can be summarized as follows: (i) After droplets come into contact with the triboelectric layer, charges (the specific type will be explained below) are induced on the surface of the triboelectric layer in contact with droplets in accordance with electric-double-layer theory. Whereas, due to electric neutrality, droplets are bound to carry other type of charge. Having been in contact with the triboelectric layer, droplets will spread, during which charges will be transferred. (ii) Droplets rebound because the triboelectric layer is hydrophobic. In the rebound process, the surface of conducting layer near the triboelectric layer has a difference of potential with the electrode, so electrons are transferred to the electrode, thus generating a current. (iii) After droplets come into contact with the triboelectric layer again while before droplets spread to maximize their contact area with the triboelectric layer, electrons flow from the electrode to the conducting layer, rebound again, and drop. Because charges on the surface of the triboelectric layer exist for a certain time. Steps (ii) and (iii) are repeated as new droplets fall^20^. Moreover, the review also explores the relationship between dropping velocity of droplets, droplet volume, and triboelectric signals (Fig. 3).

Liquids^21^ such as oil^17,22,23^, blood^24^, and seawater^8,25–29^ with certain mobility and charges can also serve as triboelectric liquids to provide charges for the entire triboelectrification system. Jin^30^ summarized the three methods of water electrification and influencing factors thereof (the liquids used in current research remain tap water, seawater, rainwater, and deionized water).

#### Temperature effects on energy harvesting

Aside from the triboelectric fluid, temperature affected the electrical output performance, and the impact of temperature differed among varied friction layer materials. This different influence was proved by experiments with S–L TENG^26,31^. First, deionized water was used as the triboelectric liquid. When using PTFE as the friction layer, the data from experiments at 80 °C were compared to the data from 20 °C; the peak voltage of PTFE at 80 °C was 1.13 times of that at 20 °C, the current was 1.05 times and the transferred charge 0.71 times. When using fluorinated ethylene propylene copolymer as the friction layer, the peak voltage at 80 °C was 2.7 times of that at 20 °C. Further, 3.5 wt% NaCl solution was used as the friction liquid. When using NaCl solution, instead of temperature, the ion concentration became the main factor affecting the electrical signal output, which may be explained by the electron cloud model and the double electric layer model^32^. Moreover, the joint effect of temperature and pH on friction power generation was studied by Zheng^33^, who proposed an energy band model to explain the tribovolatic effect of temperature on the interface of liquid and semiconductor, and verified the concept of “bindington”. These studies about temperature effect may provide novel methods to improve the energy efficiency of friction power generation.

### Triboelectric layer

The triboelectric layer is an important component of a TENG, and its polarization characteristics are critical factors for generating the difference of potential and the current. Exiting research on the topic mainly includes the following directions: finding high-polymer materials with better polarization and higher charge density from existing materials, or creating a high polymer with better experimental results through polymerization; using particle doping to introduce some particles that are difficult to attach while improving the charge density of triboelectric layers or enlarging the surface potential well, thereby increasing the amount of electrons retained on the surface, thus improving the output power; from the surface characteristics of triboelectric layer and combining with bionics, finding new manufacturing technologies of surface structures, thereby enlarging the contact area between triboelectric layers and droplets without changing the super-hydrophobicity of the surface of triboelectric layers^34^.

Taking an overview of the current research based on the surface structure of friction power generation layer, the main optimization trend focuses on improving its hydrophobic property. This strategy can effectively increase the contact angle of the droplets, thereby reducing the surface energy, decreasing the adhesion of the liquid, promoting the rapid movement of the droplets, and ultimately indirectly accelerating the accumulation and transfer rate of charge.

#### Materials for triboelectric layers

The energy generation process of S–L TENG relies mainly on the contact between the liquid and the triboelectric layer and the charge exchange between the two parties. In this process, the properties of the liquid have been discussed, while the material selection of the triboelectric layer is a key factor in determining the strength of the electrical signal. The selection of materials for the triboelectric layer requires a combination of the following: hydrophobicity, dielectric constant, mechanical properties, and chemical stability. These factors together affect the effectiveness of the triboelectric layer material, which in turn determines the performance of the S–L TENG.

##### Synthetic triboelectric materials

**Figure 4:**
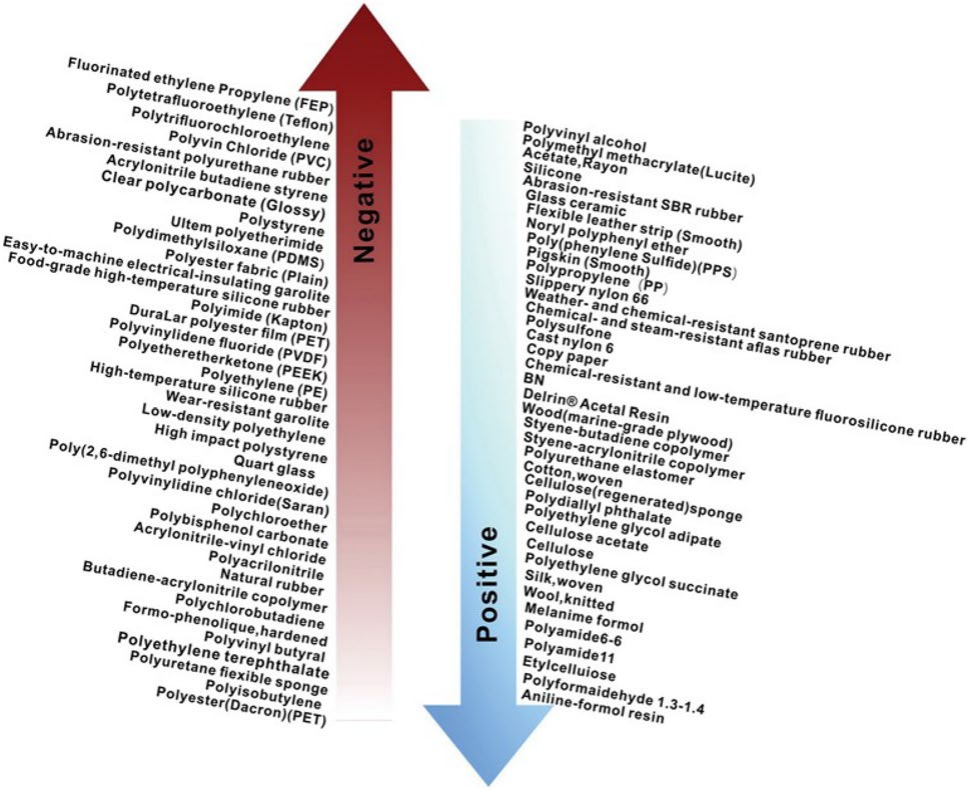
Triboelectric generation materials by positive and negative tribopolarity^35^.

Materials selected for the synthetic triboelectric layer of TENGs are often insulators. According to the ability of different triboelectric materials to gain and lose electrons, it can be seen that polymers (e.g. Teflon, polydimethylsiloxane (PDMS), and polyvinyl chloride (PVC)) are generally selected as triboelectric materials (Fig. 4)^35^. X-ray electron spectroscopy has been introduced to observe SiO_2_ surfaces modified with perfluorododecyl, oxypropyl, aminopropyl, and pure dodecyl. Final results show that the SiO_2_ surface modified with aminopropyl is detected to have positive charges, while negative charge density of SiO_2_ modified with perfluorododecyl is far higher than those modified with oxypropyl and pure dodecyl. Therefore, it can be concluded that negative charges are easily generated if the polymers contain fluorine functional groups; while positive ones are likely generated if the polymers contain amino functional groups^36^. Of course, the triboelectric layers with different chemical formulas also differ in the ability to gain and lose electrons due to discrepancy of the functional groups. The authors studied the ability to gain and lose electrons of the polypropylene (PP) film (–CH_3_), polyvinyl alcohol (PVA) film (–OH), PVC film (–Cl), polyethylene (PE) film (–H), polyvinylidene fluoride (PVDF) film (difluorinated groups), polytetrafluoroethylene (PTFE) film (tetrafluorinated groups), and fluorinated ethylene propylene (FEP) film (trifluorinated groups)^16,37^. They found that the functional groups are listed (in ascending order) as: –CH_3_, –H, –OH, –Cl, and –F according to their electron-withdrawing (EW) abilities (energy supply). The research also assessed the effects of the unsaturated groups (carbon–carbon double bond C=C) on triboelectrification and concluded that, among functional groups of the PEFE film, unsaturated groups containing C=C bonds (–CF=CF_2_ or –CF=CF–) show stronger ability to lose electrons compared with normal functional group –CF_2_.

According to statistical results of existing research, materials used as the triboelectric layer mainly include PDMS^38^, FEP^8,39^, Cytop (fluororesin)^12^, and thermoplastic polyurethane (TPU)^40^. Therein, PTFE was first proposed by Wang and has also been the most widely-used material in triboelectric layers^40–46^. In addition, based on the heat-sensitive property of *N*-isopropylacrylamide (NIPAM), some research^47^ also prepared a special material, the energy harvesting efficiency and electrical signal of which change with temperature. This provides a new research direction leading to applications in high-sensitivity temperature sensing.

Meanwhile, these dielectric materials can also be handled in special ways^48,49^, so that their output electrical signals are far better than those of untreated materials. In addition, appropriately increasing the thickness of dielectric materials^50^ can also improve the energy harvesting efficiency. Ultraviolet (UV) irradiation^51^ or thermal activation^52^ can also alter the upper limit of the surface charge density on triboelectric layers, thus influencing the contact electrification performance of dielectric layers. Considering that leaves are also super-hydrophobic and enchylema in fresh leaves can also function as conductors, some researchers also found currents are generated by leaves after modification^53^. Researchers also found that silk is not only natural and has high flexibility, but also it meets the condition for S–S triboelectric nanogeneration. In the meantime, some cellulose, chitosan and fish-skin are all potential dielectric materials^54–56^. Patel^57^ introduced a special self-healing material that can be used as the triboelectric layer for triboelectrification and exhibits favorable mechanical properties and viscosity. Based on poly hindered urea (PHU) networks, the material is formed by polymerizing polydimethylsiloxane (PDMS)-based diamine (PDMS-DA) and polyisocyanate prepared without catalysts on the networks.

**Figure 5:**
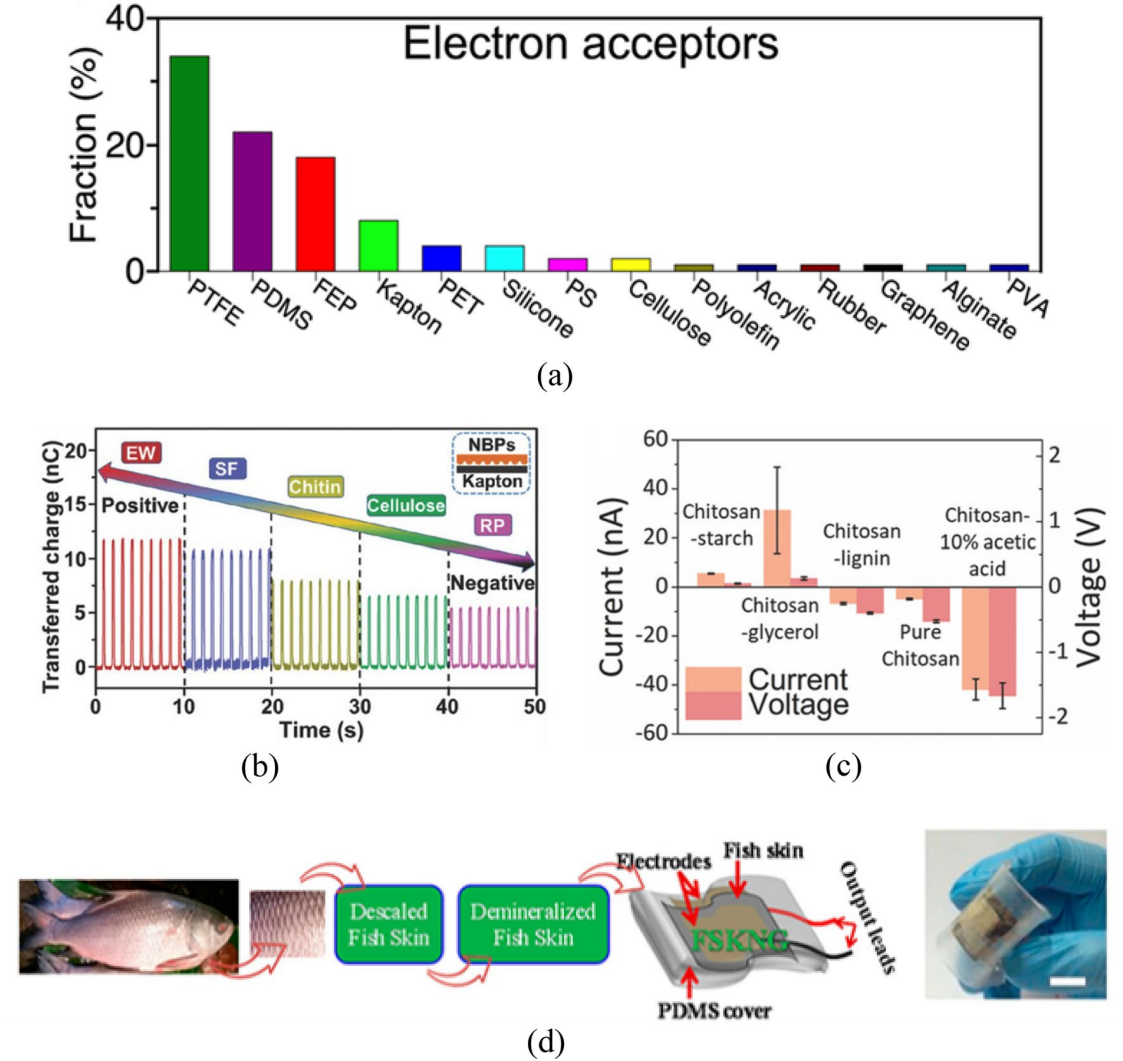
(**a**) Proportion of triboelectric materials used in 100 randomly selected articles from 2012 to 2020^58^. (**b**) Comparison of charge transfer capacity of BN-TENG prepared from different natural materials^59^. (**c**) Open-circuit voltage and short-circuit current generated when nanocomposite films composed of chitosan and different substances are in contact with Kapton films^60^. (**d**) FSKNG production process^61^.

The prevalence of PTFE as the preferred material is significantly reflected by the fact that polytetrafluoroethylene (PTFE) is mentioned in 34% of the one hundred papers on triboelectric nanogenerator (TENG)-related research. This high frequency of selection is attributed to PTFE as an outstanding electron acceptor material, while the selection of other materials is adjusted according to the specific differences in experimental requirements, demonstrating the diversity of material properties and application-specific needs in TENG research (Fig. 5a)^58^.

##### Potential natural triboelectric materials

Existing materials in nature provide a vast research space for the preparation of sustainable and biodegradable triboelectric nanogenerator. Bioabsorbable natural-materials-based triboelectric nanogenerators (BN-TENG) is a fully bio-absorbable triboelectric nanogenerator. In this paper, cellulose, chitin, silk fibroin (SF), rice paper (RP), and egg white (EW) were prepared as triboelectric layers by different methods and their piezoelectric properties were investigated. The final “triboelectric series” of these five materials were ranked from positive to negative as EW > SF > chitin > cellulose > RP (Fig. 5b). The maximum open-circuit voltage, short-circuit current, and power density generated by BN TENGs prepared with the above materials were 55 V, 0.6 μA, and 21.6 mW m^−2^, respectively^59^. It was also noted in the article that the chitoskeleton, a biopolymer derived from chitin, can be used to prepare nanocomposite membranes with flexible and recoverable properties (Fig. 5c). This nanocomposite film uses natural materials such as starch, lignin, glycerol, and acetic acid as additives to tune its properties. The surface properties are modified by laser technology to improve the performance of (TENG)^60^. The chitosan-10% acetic acid film was tested to produce an open-circuit voltage of 13.5 V and a short-circuit current of 45 nA in contact with a kind of silicone rubber (Ecoflex). In addition to this, there is a fish skin-based nanogenerator (FSKNG) with an open-circuit voltage of 2 V and a short-circuit current of 20 nA at an external pressure of 1.8 MPa (Fig. 5d)^61^. Piezoelectric sensors prepared using fish skin have a sensitivity to the outside world of 27 mVN^−1^ and a response time of 4.9 ms, and can be used as wearable devices for detecting physiological signals from the human body such as arterial pulses and vocal cord vibrations without an external power source. However, the above materials have weak electrical signals and are rarely used in S–L TENG.

##### Feasibility analysis

Materials can be categorized according to dimensionality into zero, one, two and three dimensions^62^. Zero-dimensional materials, such as carbon dots^63^ or dispersed particle dispersions of PTFE^21^, have tiny sizes and high specific surface areas. By combining these zero-dimensional materials with electrochemical properties and dielectric materials, composite triboelectric surfaces can be prepared with higher performance than pure dielectric materials. One-dimensional materials, such as carbon nanotubes^64^ or silver nanowires^30^, are flexible. Doping them into raw materials can be used as efficient triboelectric materials or carrier transport channels. Two-dimensional materials, such as graphene^4^ or plating^65^, have single or multiple layers of atomic structure. They are commonly used in triboelectric nanogeneration (TENG) to conduct electricity or to enhance properties such as hydrophobicity of the surface of the triboelectric layer. Three-dimensional materials have a more three-dimensional structure than zero- to two-dimensional materials, such as films of polymers^38^ and acrylic sheets^34^, which are commonly used as triboelectric layers or substrates in TENG.

#### Particle doping

The aforementioned research simply utilizes one material, for which the traditional method, namely, etching, is adopted^66,67^. Whereas in experiments, to meet different demands, two or more types of high-polymer particles are combined to prepare a composite triboelectric layer which has better performance in some aspects.

Multi-walled carbon nanotubes (CNTs-010-0) and PDMS dispersion liquid are fully mixed in^64^, and then stirred at 120 °C for 10 min, followed by mixing with a coagulant at the ratio of 10:1. Afterwards, 5.5 g of the mixture is allowed to spread naturally on a horizontal plane at 60 °C for 2 h. Then, a layer of insulator film is formed, which forms a film used as the electret on triboelectric layer with the output power of 1.549 mW and power density of 0.968 W/m^2^ under the load of 10 MΩ. By mixing PVA solution (10.7 wt%) with PEI solution (50 wt%), followed by mixing with the prepared solution of carbon dots (0.5 mg/mL), a highly-transparent, super-hydrophobic, corrosion-resistant composite dielectric film with a long service life can be formed after drying the prepared solution on the substrate at 80 °C for 1 h. The film can be used to combine with solar power generation^63^. Previous research also functionalizes graphene oxide with 1H, 1H, 2H, and 2H-perfluorooctane triethoxysilane to obtain powders, which are mixed with PVDF solution. The acquired solution is then used to cover an ITO film at 80 °C for 12 h. The resulting surfaces of the triboelectric layers have significantly increased polarization performance compared with the pure PVDF surface (β phases reach 83.8%), of which the dielectric constant increases from 10.4 to 32.3. Additionally, the current and voltage outputs are separately 18.1 μA and 16.5 V^68^.

#### Surface geometries

Apart from the selection of triboelectric layers that ensures charge density, the hydrophobicity and the variation of contact area between the droplets and triboelectric layers are also important indices that affect triboelectric signals. Optimization of the surface geometry of triboelectric layers is also a strand of the research. At present, the basic surface geometry is a plane that can ensure the contact surface between triboelectric layers and droplets as smooth as possible. This guarantees that droplets have an appropriate surface area after coming into contact with, and spreading over, the triboelectric layers. However, subsequent research has found that some special methods can be adopted to treat the surface of triboelectric layers, resulting in an improved hydrophobicity and larger variation of the area of droplets in the process from spread to rebound, it also reduces the residence time of droplets on the friction surface and shortens the interfacial renewal cycle^69^.

**Figure 6:**
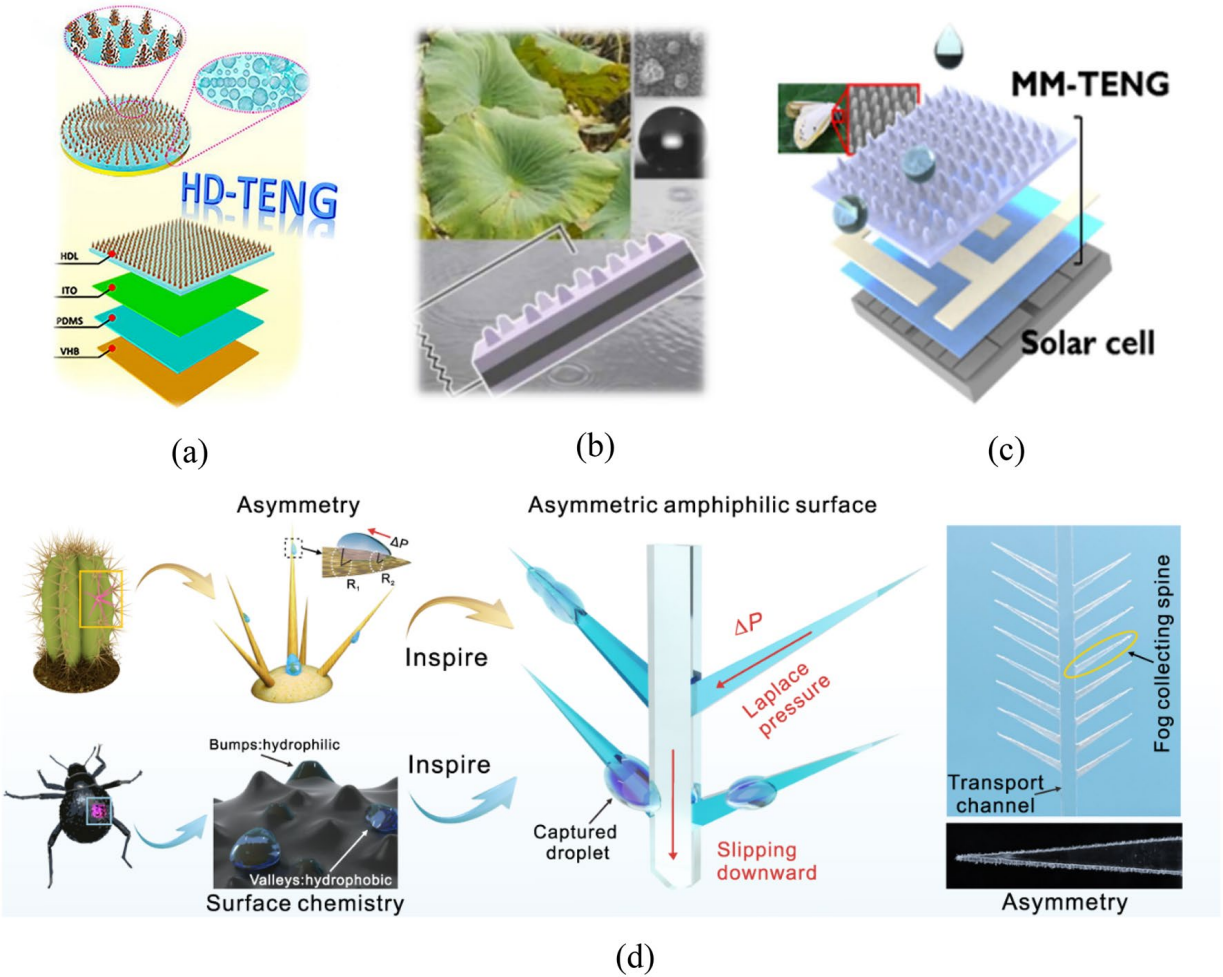
S–L TENGs with innovations in the surface geometry. (**a**) Surface geometry with the fibrous triboelectric layer^71^. (**b**) Lotus-leaf-inspired surface geometry^13^. (**c**) Moth-eye-inspired surface of triboelectric layer^73^. (**d**) Main structures of the D-TENG^74^.

At present, many scholars have proposed new surface geometries. For example, a surface geometry with randomly distributed step-shaped pattern is formed by chemical etching of substrates, removing all surface residuals, and then spin-coating the dispersion liquid of fluorine-containing groups on etched surfaces using a spin coater^70^. By fully mixing graphene oxide with the solution of PDMS precursor via magnetic stirring, a liquid film of about 500 μm can be obtained by spin-coating and curing the mixture on a glass substrate measuring 5 m × 5 m. Thereafter, a spray gun is adopted to spray the mixed liquid of methylbenzene, PDMS precursor, and magnetic carbonyl iron particles on the glass substrate cured on a magnet for drying and curing. Finally, a layer of fluoropolymer dispersion liquid is sprayed thereon to form the surface of triboelectric layers with fibrous structures^71^ (Fig. 6a). By impressing surface characteristics of lotus leaves on a FEP film through stamping and nano-hot embossing, a lotus-leaf-inspired surface of triboelectric layers with similar surface characteristics to lotus leaves can be obtained^13^ (Fig. 6b). Li^72^ also prepared a lotus-leaf-inspired surface by using the template method, which shows favorable self-cleaning, flexibility, and electrical output performance. For the electrodes, laser-induced graphene (LIG) is adopted. At first, plasma etching is utilized to etch nanocolumnar arrays on the surface of the glass substrate, which is then covered with polystyrene nanoparticles. These polystyrene nanoparticles are then treated by oxygen plasma; hydrogen and tetrafluoromethane are adopted for plasma treatment of the nanocolumn array structure on the glass surface. Finally, the surface chemical residuals are washed away to acquire the moth-eye-inspired substrate and surface of triboelectric layers^73^ (Fig. 6c). In^74^, the D-TENG combines characteristics of cactus and elytra surface of desert beetle. In this way, an asymmetric surface structure used for water storage and droplet-based triboelectrification is designed. The surface of its bumps is covered with an amphiphilic cellulose ester coating (ACEC) to collect droplets, while the stem is hydrophobic, which is used for triboelectrification (Fig. 6d).

### Electrodes and conducting parts

#### Electrode materials

The current generated by a TENG is extremely weak because of the low charge-conversion efficiency and a small number of surface charges. In view of this, it is imperative to search for better conductive materials and more efficient packaging technologies. Initially, Wang assumed it possible to supplement charges from the ground, while as the industry develops, Xu proposed to add an external electrode to the system, which can significantly improve the output efficiency. Since then, the power of S–L TENGs has been improved enormously. Statistical data from this review also show that the electrical signals output by the S–L TENG system with an added electrode are indeed better than those output by the system directly acquiring charges from the ground. The conducting layer and electrode are the same part in some TENG systems, so this part is not discussed individually. At present, main conductive materials used include graphene^9,75^ aluminum^70,71,76^, indium-tin-oxide (ITO) thin film^68,77^, copper^76,78–80^, and silver nanowires^73^. Graphene, a two-dimensional material consisting of a single layer of carbon atoms tightly arranged in a hexagonal honeycomb structure, is recognized as one of the world's hardest nanomaterials. Its remarkable properties include excellent electron mobility, high transparency, and outstanding electrical and thermal conductivity. Notably, the electrical conductivity of graphene can be modulated in a number of ways, such as by doping to introduce impurities, changing the number of layers and their stacking, applying physical strain, or adjusting the environmental conditions under which it is exposed^81^. ITO films, which are composed of oxides of indium (In) and tin (Sn), combine transparency, electrical conductivity and chemical stability. However, given the scarcity of indium, the cost of the material is relatively high. In addition, ITO films exhibit a certain degree of brittleness, which limits the range of applications in which they can be combined with flexible materials^82^. Silver nanowires, a nanoscale silver material with an extremely fine wire-like structure, have been widely used in many fields, especially in electronics, optics and materials science. The material is known for its excellent electrical conductivity, high light transmittance, good flexibility, and remarkable antimicrobial properties, especially in the development of flexible products and biomedical applications, silver nanowires show great promise^83^. For electrode applications in triboelectric nanogeneration technology, traditional metallic materials such as aluminum and copper are often found in the form of aluminum or copper foils, and the design takes the configuration of strip, sheet or inserted finger electrodes.

#### Electrode shape

**Figure 7:**
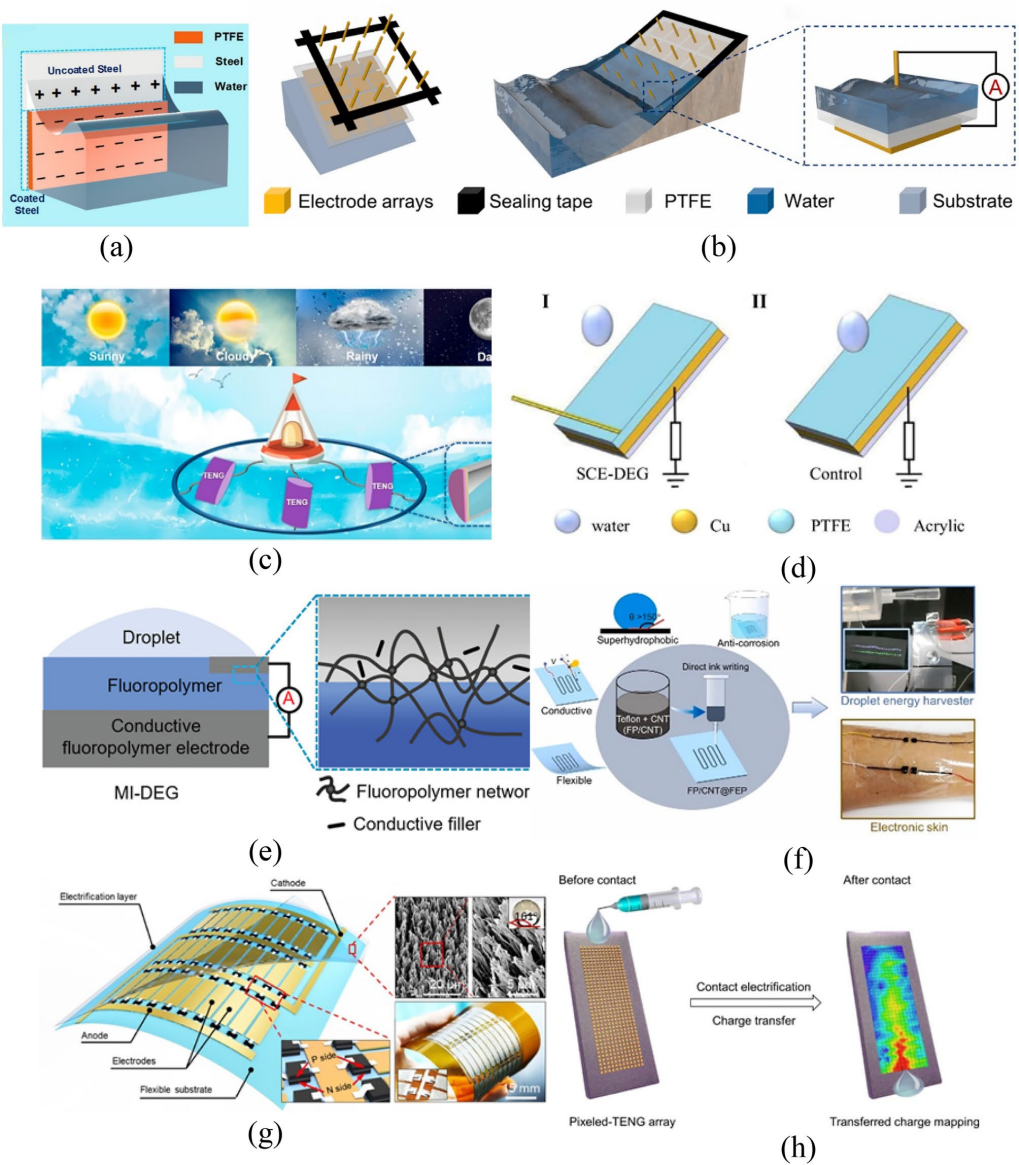
S–L TENGs with innovations in the conducting part. (**a**) Cathodic protection by regarding the ship as an electrode^26^. (**b**) Improving the output performance of the TENG using the array electrode^28^. (**c**) A closed, parallel S–L TENG system^27^. (**d**) The SCE-TENG formed by connecting different electrodes in the upper and lower parts, which demonstrates ultra-high instantaneous output power^45^. (**e**) The MI-DEG formed by hot-pressing PFA conductive pellets and the triboelectric layer together^50^. (**f**) Drawing the electrode in any pattern through DIW^84^. (**g**) The TENG formed by depositing P–N junction rectifier chips on two ends of each conducting element and connecting multiple elements in parallel^66^. (**h**) Taking copper probes as the electrode to map the variations in the motion trajectory and shape of droplets^76^.

TENGs which are regarded as self-powered equipment can provide cathodic protection for ships in the ocean and large metal-containing appliances^26^. Some researchers proposed coating a PTFE layer on the ship surface. This not only renders the ships more hydrophobic, thus reducing the drag thereon, but may also be used for triboelectrification by virtue of charges in the ocean and mechanical power brought about by wave-impact against such ships. Triboelectrification provides a steady flow of electrons for the metal layer of ships (Fig. 7a), which plays a role in avoiding corrosion of ships by seawater. In the paper, it is pointed out that the hull metal is divided into the friction power generation part coated with PTFE and the ordinary part not coated with PTFE. Wave rubbing on the PTFE surface will change its negative charge, while the positive charge inside the metal hull will transfer between the coated PTFE and the uncoated PTFE to generate an electric current, which is similar to the principle of cathodic protection by applied current. Therefore, in a wet environment, by coating PTFE will help to protect the ship.

The electrodes and conducting parts also differ in the shape in different TENGs. In some TENG systems, electrodes are added on the upper and lower surfaces of the triboelectric layer. The upper electrode is an electrode in the three-dimensional (3-d) array, which can tremendously enhance the volume effect and therefore improve the output performance of generators. The output open-circuit voltage and short-circuit current can separately reach 42 V and 4 mA (Fig. 7b). The lower electrode is mainly used for charge transfer throughout the process. Sun^27^ prepared a closed, parallel S–L TENG system composed of multiple PTFE films and water, and finally connected the circuit thereof to the load through a rectifier bridge (Fig. 7c). A special graphene specimen can be prepared by laser induction. By adding a quasi-transparent FEP film in the middle in the LIG process, fluorine can be projected onto the graphene surface. The ability to gain and lose electrons of the special fluorine-containing graphene is superior to that without the FEP film while inferior to that dosed with PDMS. In most TENGs, a circuit is formed by connecting the upper and lower electrodes via conductors, to which the load is then connected. Li proposed a droplet-based electricity generator with a simple open structure (SCE-DEG)^45^. Electron transfer on the upper surface in the S–L TENG depends on the upper electrode, while that on the lower surface is based on connection of the lower electrode with the ground and load. The ground provides a steady flow of electrons needed for transfer for the PTFE film, and the ultrahigh instantaneous output power can be realized via the self-capacitance effect of the upper electrode. Li also studied the relationship between the upper electrode length and the output power of the TENG system (Fig. 7d). Wang^50^ proposed a non-metal electrode, in which the PFA conductive film is formed by hot-pressing PFA conductive pellets under high pressure, of which the electric conductivity can reach 2.17 S/m. Compared with traditional metal electrodes, it features better ductility and corrosion resistance as well as super-hydrophobicity (Fig. 7e). Yang^84^ introduced a hydrophobic electrode material with stable chemical properties based on fluoropolymer and carbon nanotube (FP/CNT). The material can be used to draw electrodes in different patterns with robustness and high electrical conductivity on PTFE films through direct ink writing (DIW). The DEG prepared using the electrode also shows favorable output of electrical signals (Fig. 7f).

For TENGs that allow triboelectrification using rainwater or seawater, the output is mainly an alternating current (AC). To enable combined generation of multiple generating units, a rectifier bridge needs to be added to each generating unit. By simultaneously depositing P–N junction rectifier chips on the two ends of electrodes and then connecting various conductive units in parallel, which are coated with a PTFE film to serve as the dielectric material, Zhang^66^ formed a TENG formed by connecting multiple generating units in parallel (Fig. 7g). The droplet-TENG developed by Zhang also has an array electrode, while it does not aim to generate energy and harvest droplet energy relative to the above device^76^. The device is composed of 432 copper probes that are used as the electrode to penetrate the polymethyl methacrylate substrate and a FEP layer on the top to function as the triboelectric layer. The size of each array element is 400 μm, which can map dynamic information including the motion trajectory and shape variation of droplets in the array (in real time) by mapping different changes in the number of transferred charges as the droplets slip (Fig. 7h).

### Overall shapes of S–L TENGs

The shape of the S–L TENG depends, on the one hand, on its application scenario, while on the other hand, it is directly related to the mechanical properties of the fluid and the contact area with the friction layer, which is also one of the main factors affecting the final electrical energy output. Since the invention of the first S–L TENG, the internal structure has also been constantly reformed from the initial planar TENG proposed by Wang with wire grounding to obtain charges from the ground^85^ to a cylindrical S–L TENG to meet the simultaneous needs of water storage and charge trapping^86^; to a tubular TENG^87^; to rod TENGs that mainly play a role in cathodic protection in the ocean to avoid marine corrosion of machinery therein^27,47,88^; to cylindrical TENGs^49,86,89–91^; an annular TENG^22^; arcuate TENGs^92^; and a U-shaped TENG^93^. According to different shapes, TENGs have different applications, while existing research mainly focuses on sandwiched S–L TENGs that aim to harvest energy.

**Figure 8:**
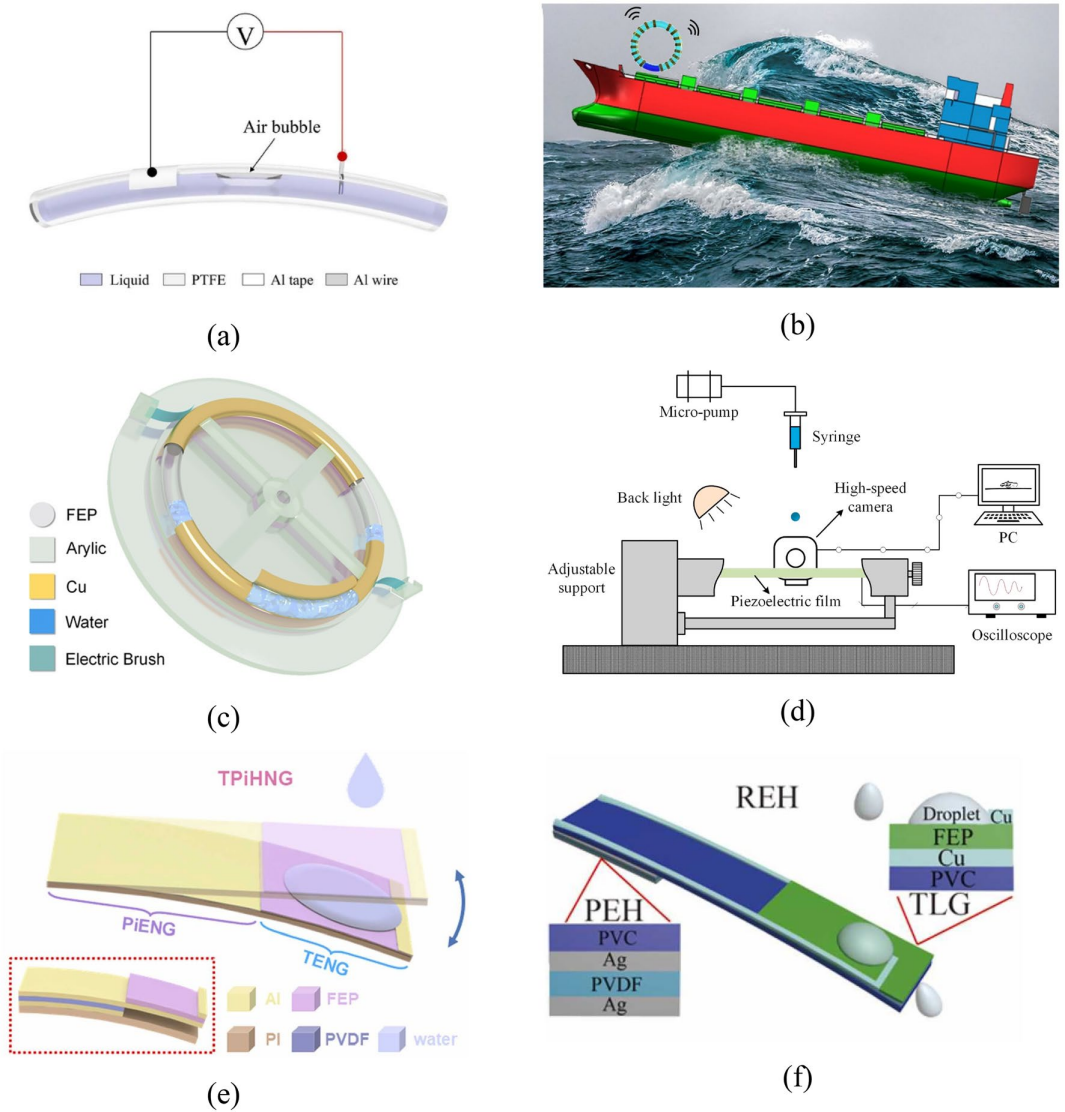
S–L TENGs with innovations in overall shapes. (**a**) A tubular S–L TENG with the fluorinated high-polymer dielectric material as the triboelectric layer^87^. (**b**) An annular S–L TENG that shows the corresponding swing angle on a ship^22^. (**c**) A DC-TENG that allows DC output by unidirectional motion of droplets in the ring^94^. (**d**) Experimental system diagram of a simply supported beam structure S–L TENG^95^. (**e**) ^96^ and (**f**) ^97^ Two S–L TENG systems with a cantilever beam structure.

For TENGs to harvest blue ocean energy, they are mainly tubular, closed structures, in which the liquid is seawater or deionized water. For the tube, fluorinated high polymers are generally selected as the dielectric materials. The liquid in such system cannot move unless mechanical energy is applied externally, so tide and wave actions are the first choices of sources of dynamic power. Such TENGs are commonly applied to the sensing field (Fig. 8a). The annular S–L TENGs are generally constituted by the annular tubes manufactured by dielectric materials (mainly PTFE or FEP), liquid sealed therein, and the electrode encircling the outer tube wall^22,94^. These two structures differ in the following aspect: the working principle introduced by Wang Song is that when a ship swings, the liquid in the annular tube rubs against the internal tube wall; because the triboelectrification effect is related to the intensity of friction between the liquid and dielectric material, the current generated during violent swinging reaches the threshold, thus raising the alarm (Fig. 8b). In the DC-TENG proposed by Wang Jiyu, a motor is used to drive rotation of the annular FEP tube, which generates friction with the deionized water sealed therein; because the motor rotates unidirectionally, this avoids the droplets spreading and rebounding multiple times on the same generating unit, therefore, a direct current (DC) is generated by the DC-TENG (Fig. 8c). Moreover, structures similar to various beams are also adopted, which can convert the potential energy accumulated in the dropping process of droplets into kinetic energy for piezoelectric power generation between two layers of dielectric materials. In addition, the process from contact of droplets with the piezoelectric material, to formation of a liquid film after spread of droplets, and to rebound and slipping of droplets can be used for S–L triboelectrification. Hao proposed a structure composed of a simply supported beam and studied influences of the dropping position of droplets on the simply supported beam, beam length, dropping height, and vibration frequency of the beam on the energy-harvesting efficiency (Fig. 8d)^95^. Aiming at a TENG with the cantilever beam structure and specially prepared triboelectric layer, Zhang not only explored influences of the dropping frequency, type, volume, and dropping height of droplets on energy-harvesting performance but also simulated the stress on the cantilever beam (Fig. 8e)^96^. Xu introduced a leaf-inspired TENG system termed a ‘rain energy harvester’ (REH) (Fig. 8f)^97^, which harvests energy through S–L contact and vibration of the cantilever beam. The system mainly includes two parts: a piezoelectric energy harvester close to the fixed end and a S–L triboelectric nano-generating part distant from the fixed end. This differs from the previous two in the following way: the S–L triboelectric nano-generating part here is established based on DEG with the additional electrode proposed by Xu, which has an external copper electrode that affords higher energy-harvesting efficiency.

### Addition

#### Other devices

**Figure 9:**
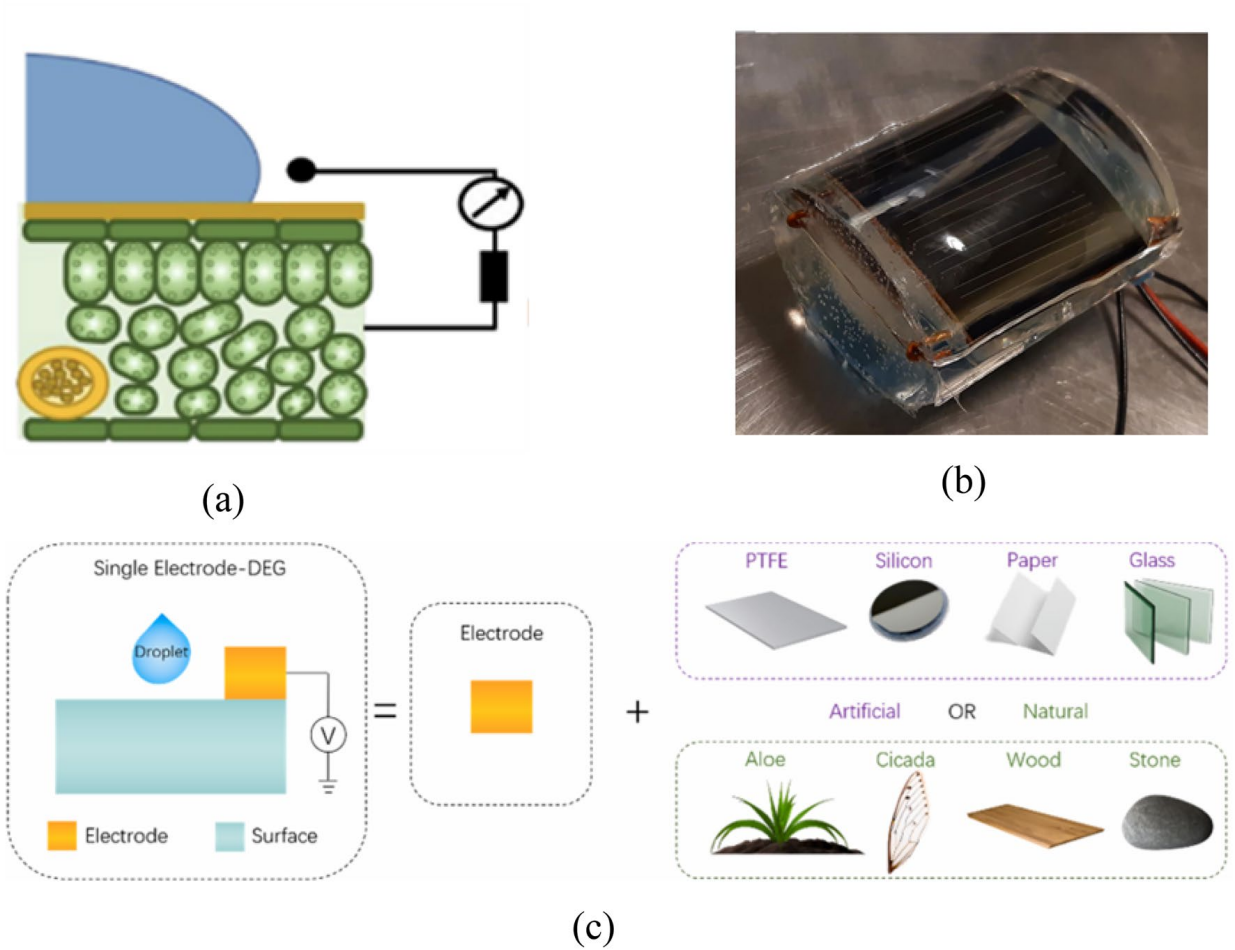
(**a**) The structure inspired by use of a leaf as the main S–L TENG^53^. (**b**) A picture of S–L TENG combining the convex lens principle with photovoltaic cell preparation^98^. (**c**) A picture of a single electrode structure using different materials for droplet electricity generation^99^.

Although other types of droplet-TENGs are not incorporated in the sandwich structure consisting of the triboelectric layer, conducting layer, electrode, and substrate, they still provide charges via droplets, thus inducing the contact electrification effect. Inspired by leaves, Wu developed a flexible and biodegradable fully biodegradable TENG (FBD-TENG)^53^. The author considered that the electrolyte in tissue layers inside leaves allows charge conduction in the entire leaves; cuticle covering the leaves functions as the triboelectric layer in a S–L TENG system due to its hydrophobicity and ability to insulate. Additionally, a closed circuit is formed by separately taking silver wire and copper as the electrode and conductor (Fig. 9a). In the article, a droplet energy harvesting surface is described which is combined with a photovoltaic cell. Unlike conventional two-dimensional surfaces that rely on substrate tilting to generate potential energy to disengage droplets, the friction power generation layer surface described in the paper is combined with the convex lens principle. Specifically, the photovoltaic cell is to a barrel structure by connecting a hydrophobic membrane to a curved electrode, and the photovoltaic cell is to a barrel. Subsequently, transparent polydimethylsiloxane (PDMS) is utilized to fill the voids to form a convex lens structure. By employing the lens principle of concentrate light, not only can the droplets slide down smoothly, but also can collect light more effectively to provide more energy for the solar cell (Fig. 9b)^98^. This article describes a novel single-electrode droplet generator that can be combined with artificial or naturally occurring surfaces such as stones, cicadas, wood, glass, etc. whose surfaces have been hydrophobized. After the surface is hydrophobized, aluminum electrodes are arranged on it, which enables the collection of electrical signals of various intensities (Fig. 9c)^99^. After testing, the peak potentials and currents of different materials were 62.2 V, − 86.5 μA (PTFE), 3.0 V, − 290.0 nA (aloe), 2.1 V, − 14.6 nA (cicada wing), 1.1 V, − 4.26 nA (silicon), 1.2 V, − 49.2 nA (paper), 3.9 V, 404.0 nA (wood), 3.5 V, 436.0 nA (glass) and 6.2 V, 350.0 nA (stone).

Some of the articles described above have data on charge number, voltage, current, and power density. In view of space constraints, detailed information is not shown in the main text, please refer to the supplementary material (Supplementary Table 1) for more information.

#### Electrowetting on dielectric

Additionally, in dielectrowetting (DEW), a topic that has not received wide attention until recent years, the applications of electrowetting on dielectric (EWOD) technology on S–L interfaces and microfluidics in the field have close relationships with S–L triboelectric nanogeneration^14,100–103^. EWOD is a technique that utilizes an electric field to change the contact angle of a droplet on the surface of a dielectric material. When an electric field is applied to a conductive substrate covered with a dielectric material, the charge at the interface of the dielectric layer is redistributed, resulting in a change in the interaction between the droplet and the solid surface, which affects the contact angle^104^. A TENG-driven electro-wetting (EWOD) droplet collector consists of an EWOD device with finger electrodes and a rotating free-suspension TENG (RF-TENG) as a high-voltage power source^105^. RF-TENG is capable of utilizing ambient mechanical energy drive and generating voltage to inhibit droplet rebound, thus increasing the droplet collection rate. It was found that the droplet collection rate can reach 95% when the output frequency is higher than 30 Hz. Also, for example, in^106^, the authors fabricated a self-driven microfluidic system by integrating triboelectric nanogeneration and EWOD technology. The S–L triboelectric nanogeneration by 80 μL droplets and the dielectric layer can drive motion of 1-μL droplets. In^107^, parameters including the slipping speed of droplets in the slipping process and the front and rear contact angles, and the energy harvesting efficiency of the entire system are discussed from perspectives of electrowetting and reverse electrowetting.

## Applications

Based on the core function of energy harvesting, S–L TENG is widely used in various fields. This chapter reviews the application of S–L TENG in energy harvesting, wearable area and medical area^7,108^.

### Energy harvesting

Energy harvesting is the fundamental application of TENG. This section introduces self-generation TENG and self-powered triboelectric system based on the flow of energy.

#### Self-generation system

**Figure 10:**
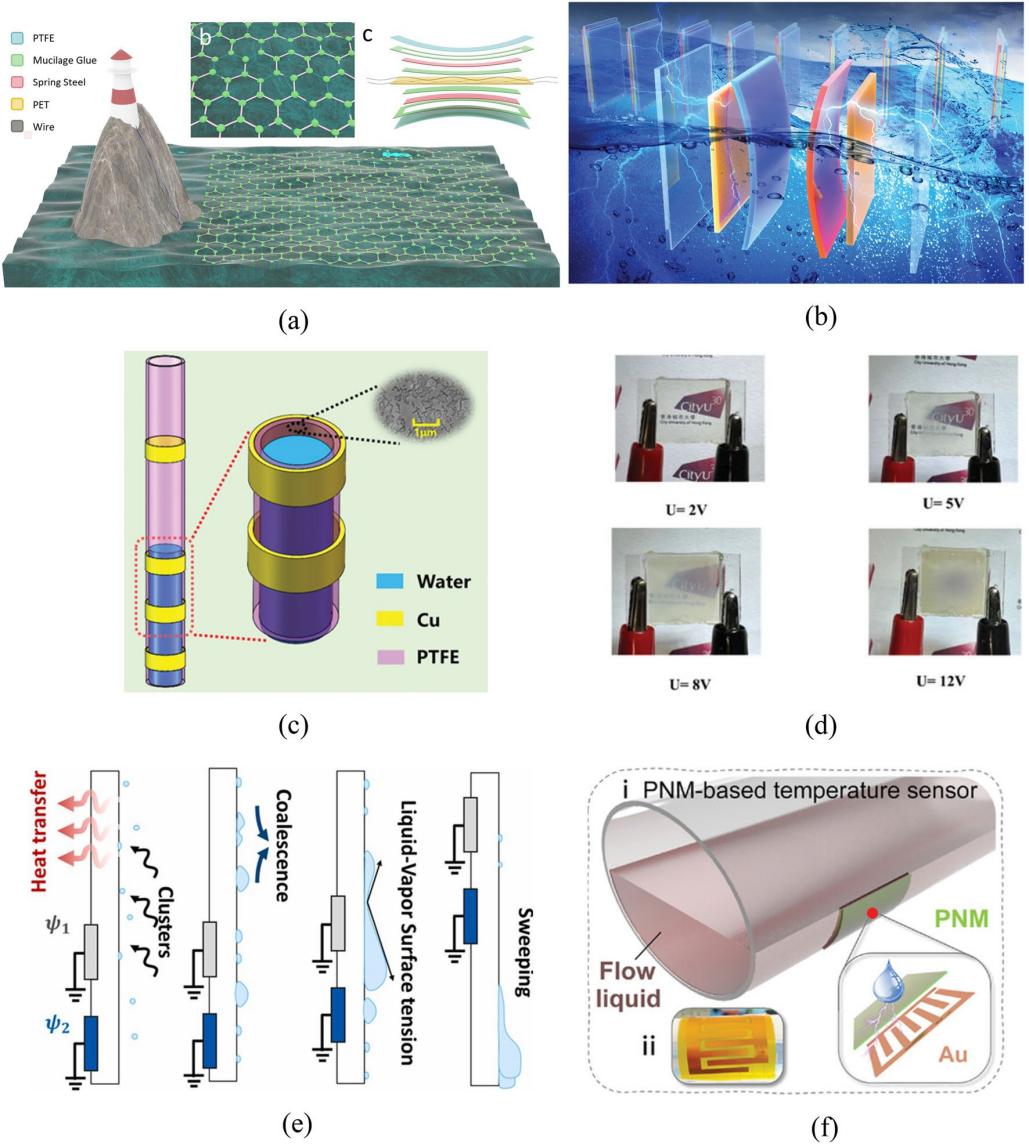
S–L TENG on applications in the area of energy harvesting. (**a**) A picture of the structure of OS-TENG and its envisioned large-scale collection of mechanical energy from ocean waves^110^. (**b**) DE-TENG asymmetric plate structure^111^. (**c**) A LST-TENG structure picture^91^. (**d**) CLC-SW obstructs vision due to electrical stimulation generated by droplet friction power generation^113^. (**e**) Steam detachment from condensation on CDEG^114^. (**f**) A picture of S–L TENG structure with PNM surface as friction layer^47^.

Self-generation TENG can be mainly classified into water-TENG and TENG using droplets to generate electricity. Water-TENG refers to TENG for ocean energy harvesting. To harvest energy, a charge transfer was produced from the friction between the irregular waves of the ocean and the friction layer. Different types of water-TENG was summarized previously by Wang et al., include S–S TENG and S–L TENG^109^. For example, the oblate spheroidal TENG (OS-TENG) used a wire rope coated with multiple layers of triboelectric layers formed by dielectric materials, which harvested ocean energy through single or multiple segments (Fig. 10a)^110^. Steel wire rope served the function of substrate to support the triboelectric generation system, and the function of electrode to conduct charge. Higher charge output could be obtained by combining multiple OS-TENGs. Likewise, the dynamic electric-double-layer TENG (DE-TENG) harvested ocean energy with an asymmetric array structure. This asymmetric structure is composed of electric-double-layers, the two layers carry positive and negative charges respectively during friction (polypropylene dielectric with positive charge and polyvinyl dielectric with negative charge), the charge transfer therefore produced electric energy (Fig. 10b)^111^.

Another type of self-generation TENG, droplet-based electricity generator, uses droplets to generate electricity. Droplets rebound from the triboelectric layer, the change of contact area between the droplets and the triboelectric layer produces electric power. By an extra electrode compared to the traditional S–L TENG, the electrical power density of DEG multiplied that of the traditional S–L TENG^11^. One example is cactus-like DEG. The spike part is hydrophilic, the backbone surface is hydrophobic and made of dielectric material. Droplets are condensed from vapor by the spike, slide rapidly along the backbone. During this process, the contact area between the droplets and the triboelectric layer changes, resulting in electron transfer and generating electric current^74^.

#### Self-powered systems

One of the main challenges in the development of current electrical devices is that traditional power supply modes are hampered by geographic location or battery life limitations, which greatly impede their ease of daily use. Therefore, the development of new self-powered devices is particularly critical^112^.

Self-powered triboelectric system can be developed upon water-TENG or DEG. One example based on water-TENG is liquid–solid tubular triboelectric nanogenerator (LST-TENG), which detects the immersion depth of a ship. LST-TENG has a thin tubular structure with inside sensors, which connect with outside copper rings that function as electrodes. Seawater flows from the copper rings to the sensors, the friction between seawater and the tube generates an electric current. When the immersion depth varies, seawater forms a circuit with the copper rings at different locations. An alarm raises when the immersion depth reaches a threshold (Fig. 10c)^91^. Based on DEG, a device to protect privacy was developed by combining a special cholesteric liquid crystal smart window (CLC-SW). DEG powers the CLC-SW, changes CLC-SW is inner structure and blurs its surface, allowing CLC-SW to block unauthorized viewing (Fig. 10d)^113^. Another device based on DEG, a condensed-droplet-based electricity generator (CDEG), can be used to detect the sudden failure of heat exchangers or coolers in factories. To do so, CDEG identified the connection between heat flux, the condensation rate of vapor, dripping and electric signal (Fig. 10e)^114^.

Other self-powered triboelectric systems were developed upon S–L TENGs. For example, a TENG array combines four electrodes of S–L TENGs to determine the spatial position of the falling droplets. When droplets hit the triboelectric layer of each electrode from different directions, different electric signals were produced. This TENG array could be applied to the inkjet printing system to monitor the position of the ink droplets^115^. In addition, a thermosensitive P (NIPAM-MMA) (PNM)-based S–L TENG was studied. First, a temperature-sensitive PNM friction layer surface—whose polymerization and wetting characteristic were affected by temperature—was prepared by free radical polymerization, spin-coating and surface modification. Further, the relation between temperature and the output electrical signal of the friction nanoelectric system was obtained, which enabled the prediction of temperature by output signal. Afterwards, the system was connected to an electrical signal amplifier. When temperatures reached a threshold, the generated electrical signals raised the alarm (Fig. 10f)^47^. There exists a self-powered sensor dedicated to monitoring the pH of liquid environments that operates on the basis of the effect of pH on the generation of triboelectric charges. At higher pH, positive ions (H^+^) promote the generation of triboelectric charges, whereas at lower pH, positive ions inhibit the formation of charges, thus generating differentiated electrical signals^116^. This S–L TENG consists of three main components: a fluorinated ethylene propylene (FEP) thin film as the triboelectric layer, four copper electrodes arranged in parallel, and a polyethylene terephthalate (PET) as the substrate. By connecting the device to a Keithley 6514 electrical measuring device, changes in electrical signals can be accurately observed under different pH conditions. The M-TENG accomplishes this by injecting different types of fluids (e.g. tap water, NaCl solutions, and CrO_3_ solutions) into a polydimethylsiloxane (PDMS) fitted with a microfluidic channel, which enables the circuitry to be connected between the PDMS, the fluid, and the bottom electrode^117^. This system allows the system to continuously monitor the fluid and its impurity levels in the fluid, avoiding the need to transfer samples to other detection systems.

### Wearable device

**Figure 11:**
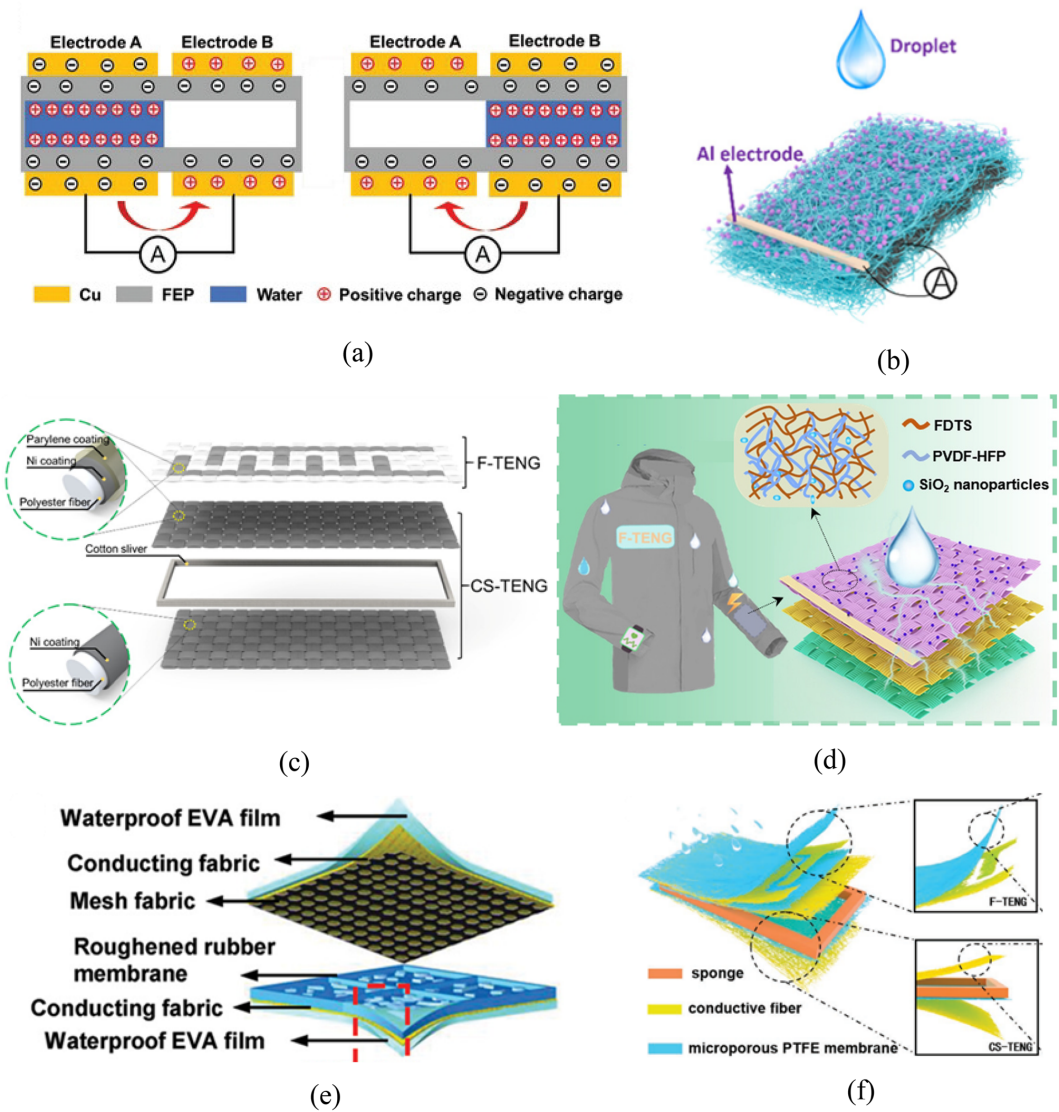
S–L TENG in the wearable area. (**a**) WT-TENG charge transfer process picture^90^. (**b**) Nanofiber membrane made of PVDF-coated e-coated carbon cloth electrode^118^. (**c**) Dual-mode textile TENG with F-TENG and CS-TENG^120^. (**d**–**f**) Pictures of three different types of laminates that can be used to make clothing^121–123^.

TENG has the advantage of small size, which facilitates its application in wearable accessories and clothing. In wearable accessories, TENG harvests the mechanical energy generated by body movement. For instance, each generator set of water-tube based TENG (WT-TENG) equals to the size of a finger with deionized water sealed inside, multiple sets could be connected and fixed on a wristband. By swinging the arm, the friction between the water and the wall of the generator produces electricity, which can power 150 light-emitting diodes (Fig. 11a)^90^.

In the field of wearable clothing, the experiment mainly adopts the electrostatic spinning process to prepare the triboelectric layer and fully combine it with the conductive layer. The special flexible material prepared by electrostatic spinning process can have good hydrophobicity and flexibility. For example, an all-fiber-based single electrode triboelectric nanogenerator (F-TENG) using electrostatic spinning technology and a coating process is a polyvinylidene fluoride (PVDF) nanofiber membrane with a built-in polydopamine (PDA) e-coated carbon cloth electrode. The open-circuit voltage, short-circuit current, and maximum power density of this F-TENG are 270 V, 80 μA, and 1.79 W/m^2^, respectively^118^. In addition, chitosan extracted from shrimp shells was used for electrostatic spinning to prepare electrostatically spun fibers with chitosan concentrations of 1.5 wt%, 2 wt%, and 2.5 wt%, respectively, and to compare the piezoelectric properties of electrostatically spun fibers with different concentrations of chitosan (Fig. 11b). The results show that the electrical output characteristics of the electrostatically spun fibers are optimal when the chitosan concentration is 2.5 wt%. In addition, a novel application is proposed in the paper, i.e., the use of such fibers in a circuit system for self-powered sensing. The system can control the start-up and shut-down of household appliances^119^. A dual-mode textile TENG comprising two TENGs is presented in the literature^120^. Contact-separation-mode TENG (CS-TENG), which is used to collect mechanical energy generated by raindrops, wind, and human motion, and freestanding-mode TENG (F-TENG), which is specifically designed to capture the energy of raindrops, respectively. Experimental results show that the F-TENG can generate up to 4.3 V and 6 μA of current under the influence of raindrops. During human motion, the F-TENG is capable of generating up to 120 V and a power density of 500 mW m^−2^. In the fabrication of freestanding triboelectric nanogenerator (F-TENG), pre-cleaned polyester fabric stuck Kapton tapes were processed using CO_2_ laser precision machining technique to keep the fabrics intact while forming interdigitated patterns on the surface. Finally, chemical deposition technology is used to form electrodes on these patterns, and parylene is coated on the surface of the fabric as a triboelectric layer to complete the construction of F-TENG. In the fabrication of freestanding triboelectric nanogenerator (F-TENG), pre-cleaned polyester fabric stuck Kapton tapes were processed using CO_2_ laser precision machining technique to keep the fabrics intact while forming interdigitated patterns on the surface. Finally, chemical deposition technology is used to form electrodes on these patterns, and the fabric surface is coated with Parylene film was a triboelectric layer to complete the construction of F-TENG. CS-TENG is composed of two fabrics, separated by a tampon, consisting of Ni-coated polyester textile and polyester textile coated with Ni and Parylene (Fig. 11c). TENG harvests the energy generated by the friction between raindrops and clothing surfaces, or between different parts of clothing surfaces (Fig. 11d)^121^. For example, waterproof and fabric-based multifunctional triboelectric nanogenerator (WPF-MTENG) is membrane-shaped. WPF-MTENG consists of two friction layers made of water-repellent ethylene–vinyl acetate, with a conductive layer and a mesh fabric layer sandwiched in between (Fig. 11e)^122^. Another type of clothing, free-standing triboelectric-layer triboelectric nanogenerator (F-TENG) comprises a woven triboelectric layer with special fiber, with a conductive layer attached. When combining F-TENG with a contact-separation mode triboelectric nanogenerator, the system could simultaneously capture the energy generated by friction between raindrops and clothing, and friction between clothing surfaces (Fig. 11f)^123^.

### The medical area

**Figure 12:**
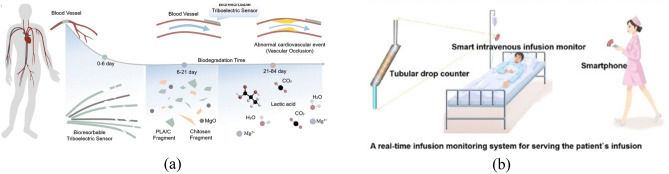
S–L TENG application scenarios in the medical area. (**a**) The S–L TENG is used to monitor blood flow and is eventually broken down and absorbed^124^. (**b**) In^24^, it mentions the application scenarios for intravenous injections.

The application of S–L TENG in medical field is less studied. A bioabsorbable friction electric sensor (BTS) could detect vascular occlusion events in vivo, was made of 4% poly (lactic acid)-chitosan and attached to blood vessels. A blockage in a blood vessel considerably changes the blood flow, which triggers a detectable signal from BTS. BTS is absorbable after five days, which eliminates surgery of implant removal and limits potential safety hazard (Fig. 12a)^124^. A droplet-based energy harvesting superhydrophobic membrane (DESm) was developed using droplet-based S–L TENG, showing antibacterial and super-hydrophobic features. The membrane mainly comprises antimicrobial nanoparticles made of quaternary ammonium silica (QSi) and a coating of polysilsesquioxane (PSQ). QSi allows for the antibacterial property by its positively charged amino groups which can disrupt bacterial membranes, and by its long alkyl chains which can penetrate the bacterial cytoplasm and destabilize the internal structure. Meanwhile, the PSQ coating allows for the super-hydrophobicity^125^. There is also a surface that is extremely hydrophobic to blood is mentioned. The surface not only has excellent flexibility and sensitivity, but can be used to monitor flow changes during intravenous injections and blood transfusions (Fig. 12b)^24^.

## Conclusion

In this review, we briefly summarize the recent advances in the factors affecting the electrical output of the S–L TENG in terms of its components as well as its operating environment. In the study of S–L TENG modules, the optimization of surface hydrophobicity, charge-carrying and transfer capacity is achieved by tuning the material and surface characteristics of the friction-generating layer, which can significantly affect the electrical energy output. In studies related to the conductive part, materials are the key factor that dominates the electrical conductivity. Different materials exhibit different charge transfer capabilities, which directly affects the electrical energy output. Also, the form or state of the electrodes is a factor in the electrical energy output of S–L TENG. In addition, the shape of the substrate can directly influence the movement of the liquid and the contact state of the friction layer, which in turn regulates the electrical energy generation process. It has also been found that when acid–base salt solutions are used as friction droplets, the charge transfer of S–L TENG first increases with increasing solute concentration, reaches a peak and then gradually decreases with further increase in concentration. In addition, the electrical signals generated by the S–L TENG of different dielectric materials are enhanced with increasing temperature, while the effect of temperature is more limited relative to the effect of solute concentration. At the same time, the droplet type and drop height regulate the electrical energy output during solid–liquid contact electrification. This review also summarizes recent advances on S–L TENG in different application scenarios. Although the research of S–L TENG has gradually spread from China to the United States, South Korea and Norway, the instantaneous short-circuit current of S–L TENG output still cannot break through the milliamperes level compared with the traditional power generation method. Moreover, due to the limitation of materials, the average electrical signal of most S–L TENGs can only be maintained in the microamp range. As a result of the research, the application scenarios of S–L TENG have been developed to energy capture, wearable devices, and medical treatment. In terms of energy capture applications, the current focus is on two directions, one is the development of self-generation devices based on liquid energy, and the other is the establishment of self-powered systems that collect liquid energy to power electronic devices. However, as far as wearable devices and medical applications are concerned, there is still room for research due to the limitations of the material properties of the friction-generating layer, the stability of the electrical energy output, and the comfort of the user. Finally, this review also suggests that further studies on the energy conversion efficiency as well as the charge transfer mechanism of S–L TENG are still needed.

## Future research directions

### Theoretical studies

At present, the majority of relevant studies are mainly experimental, while there are few studies of the underpinning principles. For example, researchers^108,126^ studied the dropping process of droplets from the dynamic perspective and found that even under the best conditions so far, the energy conversion rate from droplets to electricity is only 10%. Verification also concludes that the energy is mainly dissipated in a viscous manner by the triboelectric layer and the electricity-conversion efficiency is poor. The viscous surface was studied through molecular dynamics simulation from a microscopic perspective. For example, researchers^19^ simulated influences on the charge transfer mechanism of the dielectric layer with different ion types and concentrations via molecular dynamics simulation. The electrostatic field in the entire operation process was also investigated. For example, Tang^127^ explored the electrostatic field during the friction between the liquid and dielectric by introducing a contactless test environment and concluded that ions are main contributor for charge transfer, while the attraction between electronegativity and static electricity affects the initial charge density of the dielectric layer. Zhao^37^ introduced the generation of free radicals in the friction process between droplets and electrolyte and its influencing factors. The simulation allows for a more intuitive observation of the potential change and charge transfer rate during droplet impingement on the dielectric layer in a physical field where the multiphase flow is coupled to the electric field in both directions. The results of the simulations were verified by equipment such as Keithley 6514, oscilloscopes and nanocoulomb. However, taking COMSOL as an example, the difficulty of the current simulation lies in the fact that the bidirectional coupling of two physical fields, electrostatic and flow, cannot be realized by relying only on the software settings, and it is necessary to derive formulas such as Maxwell’s surface tension inserted into the flow field to realize the coupling. In order to make the simulation results closer to the real situation, we also need to consider the boundary conditions added in different application scenarios, which are all urgent problems to be solved.

### Direction optimization

On the one hand, enhancing the durability of S–L TENG is an important direction for optimization work. Although the currently used polymer materials such as PTFE and FEP have good chemical stability, can adapt to most chemical corrosive environments, and have relatively low wear in the occasion of triboelectric generation with water droplets, to further improve the applicability of S–L TENG under variable working conditions, the electrode and substrate However, to further improve the applicability of S–L TENG under variable operating conditions, the selection of electrode and substrate materials is particularly critical. The application of special encapsulation technologies is essential to enhance the protection of the device in different environments, which includes improving its resistance to seawater corrosion, electrostatic noise in the sensor, and maintaining its performance under extreme temperature and humidity variations. Therefore, research and development of new materials and advanced encapsulation methods that are compatible with the needs of specific application environments to enhance the durability and operational stability of S–L TENGs is also one of the current optimization directions for S–L TENGs.

On the other hand, the pursuit of higher power generation efficiency and a wider range of application scenarios is also the main direction of S–L TENG’s development. These include finding more suitable materials to produce the triboelectric layer, developing surface geometries enabling higher conversion efficiency, and more deeply exploring the underpinning mechanism to lay a theoretical foundation for future research. On the other hand, it is suggested to seek more suitable electricity-usage scenarios at high voltages and a low current. Academician Wang Zhonglin once conceived of applying the technology to cardiac pacemakers, which can be self-powered by blood and free patients from the annoyance of regular battery replacement. S–L TENGs may also be combined with other power-generation methods, as in the aforementioned combination with solar power generation^128^. This allows power output even under poor light conditions such as on cloudy and rainy days, taking advantage of their self-cleaning and high light transmittance. S–L TENGs can also be integrated with piezoelectric power generation, as TENGs with the cantilever beam structure mentioned above, or they can be added to wearable devices using certain soft, ductile, triboelectric materials. At present, among studies to find more efficient surface geometries, only a few are related to S–L TENGs. Despite this, the preparation of super-hydrophobic surface geometry has been extensively studied. In the future, S–L TENGs can probably be prepared with triboelectric nanogenerating materials while referring to the aforementioned preparation methods.

### Testing program

The testing of S–L TENG is mainly divided into two parts, on the one hand, it includes the testing of power generation performance, such as real-time monitoring of output voltage and current by connecting oscilloscopes and electrostatic monitoring equipment, evaluating the efficiency of power conversion and transmission by connecting different external loads, and observing the changes of electrical signals under different working conditions by means of sensors. On the other hand, fine characterization of the surface properties of the friction layer is also crucial. The microscopic surface structure of S–L TENG triboelectric layer can be observed meticulously by using Scanning Electron Microscope (SEM), and the crystalline structural composition of the triboelectric layer can be accurately characterized by X-Ray Diffraction (XRD) technique. In addition, contact angle measurement allows for an in-depth study of the hydrophobic nature of the friction layer surface, while the use of X-ray Photoelectron Spectroscopy (XPS) helps to reveal the composition of the surface elements and their chemical states, a process that is indispensable for understanding the process of surface modification or functionalization conversion. As seen in the supplementary information (Supplementary Table 1), there is currently variability in the description of power, including different representations of load selection as well as units in terms of power, a phenomenon that may pose an obstacle for readers in the process of horizontal comparisons. Therefore, it is recommended that future research should begin to harmonise these presentation standards.

**Figure 13:**
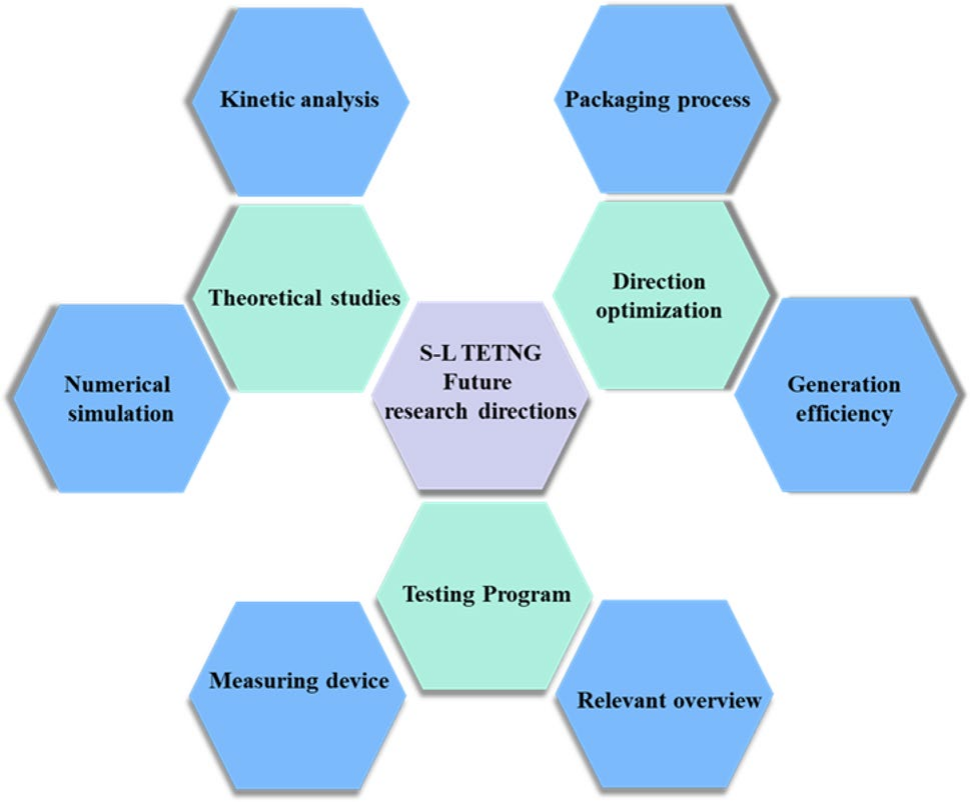
Summary chart of future research directions.

For the future research of S–L TENG, in-depth theoretical explorations and accurate simulation analyses are particularly important. Only with a deep understanding of the underlying principles, a fundamental breakthrough in liquid–solid contact triboelectric technology can be achieved. Meanwhile, the development of more efficient and stable triboelectric materials, as well as the expansion of their possibilities in more applications, are the keys to make S–L TENG technology benefit the society. In addition, improving the measurement methods of electrical signals to obtain more accurate data will help to fully understand the working mechanism and performance of S–L TENG under different environmental conditions, as well as to effectively identify and solve potential problems in operation. We expect that future research will reveal more in-depth understanding and promote the development of S–L TENG technology in the direction of greater efficiency and reliability (Fig. 13).

The core findings of this study have been discussed in section “Conclusion” and will not be redundantly elaborated here. This arrangement is intended to fit more closely into the logical architecture and enable the reader to gain a deeper understanding of the influencing factors of S–L TENG and its scope of application.

### Supplementary Information

Supplementary Information.

## Data Availability

The datasets used and/or analysed during the current study available from the corresponding author on reasonable request.
